# Transforming a Concept in a Tool: Diagnostic and Prognostic Value of Tasks Depleting Cognitive Resources

**DOI:** 10.3389/fpsyg.2021.787374

**Published:** 2022-01-27

**Authors:** Maria Silvia Saccani, Giulio Contemori, Chiara Corolli, Mario Bonato

**Affiliations:** ^1^Department of General Psychology, University of Padua, Padua, Italy; ^2^Padova Neuroscience Center, University of Padua, Padua, Italy

**Keywords:** dual-task, diagnosis, prognosis, cognitive resources, normal aging, brain injury

## Introduction

Converging evidence suggests that cognitive resources are limited and depletable. In this opinion paper we will describe how to exploit these characteristics at the clinical level. By using demanding tasks that require participants to fully engage their attentional resources (e.g., dual-tasks), it is more likely to reveal the presence of subtle motor and cognitive deficits and thus achieve high diagnostic and prognostic power. We will describe the potential this approach has for detecting and predicting cognitive deficits along a continuum from normal to pathological functioning, in apparently healthy aging as well as in neuropsychological cases. In addition, we will highlight that these more sensitive tasks are also better suited to mimic those complex everyday life contexts where patients, often unaware of their difficulties, are unable to compensate for their deficit. The case for depletion of cognitive resources as a clinical heuristic/tool is discussed.

## Implementations of the Dual-Task Method

A variety of tasks are considered particularly suitable for studying cognitive resources. These tasks, be them related to dual-tasking, multitasking, task switching or other similar settings, almost invariably require participants, broadly speaking, to be fully engaged at attentional level and thus negatively impact performance by a general reduction of available cognitive resources (Howard et al., 2020). In the context of the widely used dual-task method the difference in performance between the single (better performance) and the dual-task condition (lower performance) is called “cost” (Leone et al., 2015).

### Dual-Tasking in Normal and Pathological Aging

Studies on the impact of dual tasking are often performed on older age participants. Aging is a complex process, Harada et al. (2013) and includes normal and pathological aspects which often cannot be immediately disentangled, but rather extend along a single dimension. Since, some activities become deficient only when carried out simultaneously, categorizing performance along this continuum might sometimes be possible by exploiting dual-tasks. Such approach might become particularly informative when no clear symptoms of pathological aging are present. Two specific dual-task indexes (“Stops walking when talking” and “Useful Field of View”) provide, respectively, a precise estimate of the risk of accidental falls and of possible car accidents while driving.

#### Dual-Tasking and Locomotion: Does Aging Make It Difficult to Walk and Talk at the Same Time?

**Figure 1:**
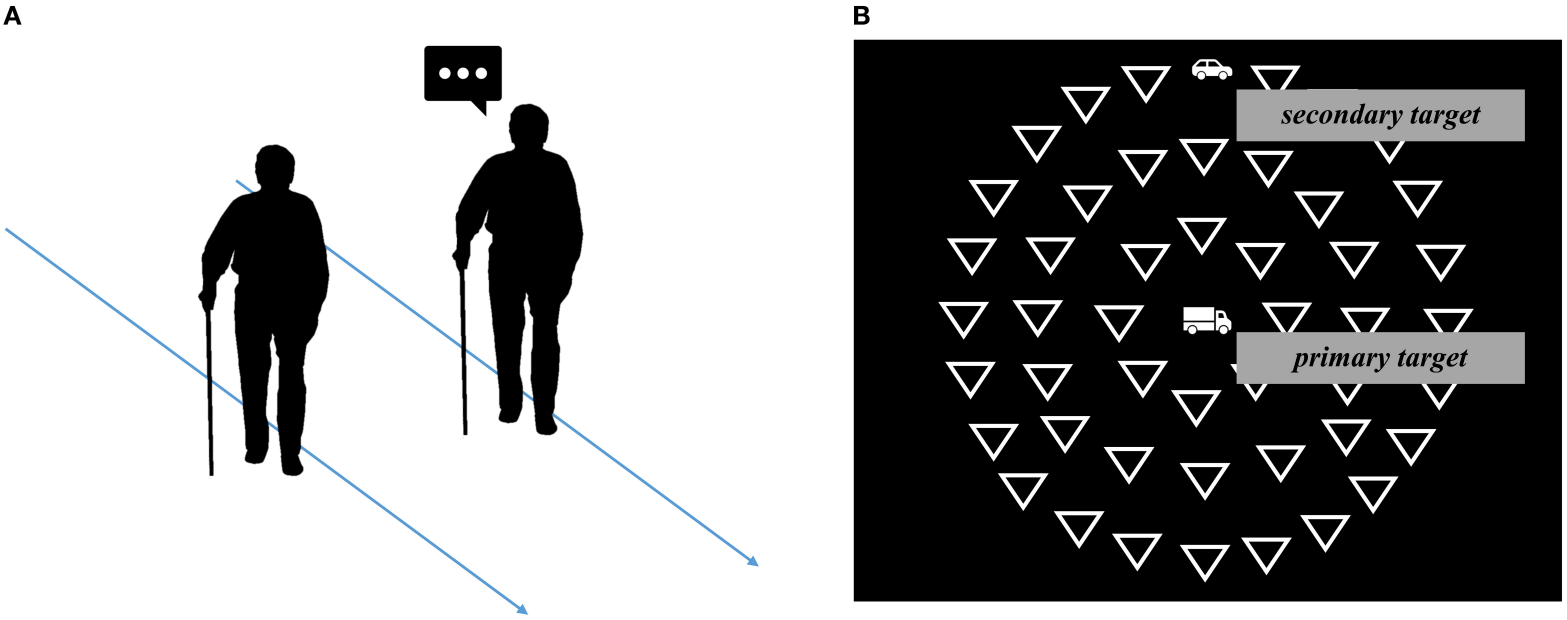
Panel **(A)** (left): The “Stops walking when talking” behavior is typically detected by asking questions and determining whether the patient stops walking while responding or manages to complete both activities together (Lundin-Olsson et al., 1997). Panel **(B)** (right): Illustrative representation of the Useful Field of View task, which consists in a central visual identification task (primary task) performed either in isolation or while paying attention to peripheral stimuli (secondary task) and ignoring distractors (Ball and Owsley, 1993).

In the motor domain, dual-task manipulations are widely used for identifying those persons more at risk of falling in everyday-life context (Schaefer, 2014). In clinical practice, a phenomenon widely known for its simplicity of detection, clinical value and predictive power is the “Stops walking when talking” behavior, or SWWT. The presence of the SWWT failure is typically detected by asking questions (e.g., about medications) and determining whether the participant manages to complete both activities together or stops walking while responding (Figure 1A). Lundin-Olsson et al. (1997) showed that the majority of older people who were unable to continue walking while talking experienced a fall within 6 months from the test, while the risk of falling for seniors who were able to continue walking while talking was significantly lower (Lundin-Olsson et al., 1997). Since then, several studies have supported the effectiveness of the SWWT test in identifying older people at high risk of falls (Verghese et al., 2002; Beauchet et al., 2009; Ayers et al., 2014).

The drop in performance found in dual-task is not limited to predictions within the broad realm of motor performance but can also extend to cognitive aspects and detect impairments related to degenerative disorders. In fact, dual-task cost allows discriminating between healthy controls, mild cognitive impairment (MCI) patients, and Alzheimer's disease (AD) patients thanks to a good association between cognitive and molecular biomarkers, and a moderate prognostic value (Nielsen et al., 2018). A longitudinal study (Montero-Odasso et al., 2017) has shown a good prognostic value of a working memory dual-task on walking parameters in MCI patients. The larger the cost the higher the probability to develop severe cognitive disorders/dementia.

In short, research using dual-task suggests that dual-tasking can be a viable option for the sensitive and early detection of subclinical motor but also cognitive deficits in ecological contexts with good diagnostic and prognostic ability. Evaluating performance in dual-task conditions makes it possible to identify potential problems “sooner and better” compared to the canonical tests, which usually are not sensitive enough to detect subtle deficits. Consequently, it might be possible to reduce the emergence of such problems in the most complex situations of daily life, where they could lead to negative outcomes; indeed, for an aged person, a fall can have very serious consequences (Kannus et al., 2005). What about other potentially dangerous situations such as driving?

#### Dual-Tasking and Visual Processing: Can We Derive From an Experiment Whether an Aged Driver Is at Risk for a Car Crash?

Driving is a complex task that can become particularly difficult for older people, who are exposed to specific risks due to motor, sensory and cognitive impairments. It is known that the need to simultaneously process two visual stimuli (i.e., a dual-task) induces a “shrinkage” of visual field, i.e., reduces the number of peripheral targets detected. A computer-based test widely used to verify this phenomenon is the “Useful Field of View” (Ball and Owsley, 1993; Sekuler et al., 2000; Edwards et al., 2006). In this test, a central visual identification task (primary task), is performed either in isolation or while paying attention to peripheral stimuli (secondary task), while ignoring distractors (Figure 1B). As in the motor field the SWWT phenomenon often anticipates a fall, the UFOV effectively predicts important everyday life outcomes, like car crash risk (Clay et al., 2005). Driving performance is significantly more impaired in those older people who present a particularly large, dual-task induced, reduction in their useful field of view (Rubin et al., 2007; Cross et al., 2009). The predictive power of UFOV might be due to its multidomain nature: the test requires not only visual processing but, when task complexity is increased, also high order attentional abilities, that are crucial when driving in traffic. This is in line with the fact that test batteries testing multiple cognitive domains, rather than vision alone, are more able to predict driving outcomes (Wood et al., 2008, 2013).

The UFOV is widely used by healthcare professionals, and the current commercial version (i.e., UFOV®. Visual Awareness Research Group, Punta Gorda, FL) requires only 10-15 minutes to be completed and can run on a personal computer (Wood and Owsley, 2014). Online versions might be developed in the future in order to reach a higher number of potentially at risk older drivers.

### Dual-Tasking as a Tool for Detecting Visuospatial Disorders in Neurological Patients

We have so far focused on prognostic aspects, whereby dual-tasks offer a particularly suitable approach for predicting performance in everyday life contexts. A further field of application of this method is diagnostic and has been successfully implemented for the evaluation of neurological patients who experience visuospatial difficulties following neurodegenerative disorders or brain injuries.

#### Closing-In Behavior Parkinson's Disease

De Lucia et al. (2015) used a verbal dual-task to test whether closing-in behavior (i.e., the tendency to draw near or on the visual model/shape that is supposed to be copied) in Parkinson's patients is exacerbated by a second task taxing on verbal, non-spatial, domain. The tendency to deviate towards the model strongly correlated with executive dysfunction, and significantly increased when patients were engaged in dual-task compared with single-task conditions (De Lucia et al., 2015). Such findings suggest a relationship between reduction of attentional resources and the presence of closing-in.

#### The Case of Deficient Contralesional Processing Following Stroke

Following a brain injury (caused for example by a stroke) patients may show a deficit called unilateral spatial neglect (Driver and Vuilleumier, 2001; Corbetta and Shulman, 2002; Bartolomeo et al., 2012; Vuilleumier, 2013), which is characterized by difficulties in processing a contralesional portion of the surrounding space (Bonato, 2012). This condition negatively impacts everyday life: right hemisphere damaged patients, for instance, can suffer injuries from hitting objects on their left. In clinical practice, the diagnosis of this disorder is carried out through specific paper tests that require, for example, to cross all the stimuli presented in a sheet. The majority of chronic patients present only mild, if any, deficits in these tests, despite experiencing difficulties in complex, everyday life situations. However, their deficits emerge again in computer-based tests (Rengachary et al., 2009). Building on the previously described clinical dual-tasking approaches we simulated, in a computerized, controlled and simplified context, a visuospatial environment requiring patients to pay attention to several aspects at the same time. Across different studies, we demonstrated that a secondary task (visual or auditory), performed during a main visuospatial task (i.e., naming whether a lateralized target appeared on the left or on the right of fixation), interferes with the perception of the contralesional space in chronic patients not showing neglect in classical tests (Bonato, 2015; Bonato et al., 2019). These seemingly unimpaired patients, while achieving good performance in the single-task conditions (i.e., execution of the visuospatial task only), lost this ability in the dual-task conditions, but only when the target was presented in the contralesional “weak” space. In striking contrast, targets presented on the non-compromised side were always perceived. In short, the left target was perceived by right hemisphere damaged patients only when they could focus on its appearance without having to pay attention to anything else. The fact that the impairment was very similar when the secondary task required to pay attention to a sound or a visual symbol provides important clues to the origin of this interesting phenomenon. One might even claim that the source of the distraction (whether a sound or an image) was not important as much as the presence of a distraction itself. In conclusion, it is possible to assume that, under the less demanding single-task condition, resources are employed to optimize an otherwise impaired performance. However, in the dual-task condition, resources were insufficient and the impairment couldn't be compensated.

The “implementation/operationalization” of the concept of “cognitive resources” in dual tasks has therefore once again proved to be a very sensitive approach in identifying subtle forms of cognitive deficits which represent a real diagnostic challenge for any clinician.

### Future Directions: Dual-Tasking Might Become a Tool Also in the Rehabilitation Field

According to a recent meta-review, dual-task training could significantly improve motor and cognitive functions (Oliva et al., 2020). A moderate level of evidence suggests indeed that both cognitively healthy and pathological individuals (patients with MCI, AD and stroke) improve after interventions tapping on memory and attention.

Ecological dual-task training was shown to specifically improve executive control in aging (Wang et al., 2011). The training was based on the breakfast cooking task (Craik and Bialystok, 2006), in which participants are required to cook several foods and concurrently set a table. During the post-training evaluation, the improvement was found to also extend to independent tasks like WAIS-III sub-tests. Other ecological dual-task trainings were shown to help older people to improve their visuospatial processing thus leading them to keep their driving license longer (De Angelis, 2009). Some of these trainings appear to be effective even in reducing the rate of road accidents (Ball et al., 2010) and in allowing to drive safely for a longer period (Edwards et al., 2009).

Dual-task training also seems to improve gait disorders in Parkinson's (Strouwen et al., 2015). Seven patients showed significant improvements in gait speed and gait variability until one month from the end of the training program (Yogev-Seligmann et al., 2012). However, a training program in neglect patients consisting of 30 training sessions across 6 weeks, and coupling a visual scanning training together with a driving simulator task did not induce positive effects (Kessel et al., 2013; but see Van Vleet and DeGutis, 2013).

In conclusion, dual-task training might become a useful rehabilitative tool, but other studies are required to corroborate the positive results already present.

## Conclusive Summary and Open Questions

Complex everyday life contexts are difficult. In more theoretical terms we can claim that they engage many of the attentional resources available. Tests based on the dual-task method (SWWT, UFOV) have an early prognostic value for older people across a variety of contexts. The use of the dual-task has also proved useful as a diagnostic tool for highlighting deficits in visuospatial processing in neurological patients, while its rehabilitative potential is still uncertain. Additionally, it remains to be defined whether dual task cost correlate with other, important sources of information such as biomarkers. The clinical implications of the dual task approach go beyond the increased sensitivity and concern the correspondence/analogy between the laboratory test and the complex situations of daily life where patients, often unaware of their difficulties, are unable to compensate for their deficit.

Altogether, evidence suggests that the overall amount of resources is a major determinant for performance. We, therefore, maintain that resource-demanding tasks can be useful for clinical purposes and that the concept of resources can be very informative when applied as domain-general.

## Author Contributions

All authors were equally involved in conceptualizing, preparing the draft, and editing the manuscript.

## Funding

MB was supported by a STARS (164480) grant from Unipd and by a PRIN grant from Italian Ministry of University and Research. This paper was carried out within the scope of a Use-inspired research project, for which the Department of General Psychology of the University of Padova has been recognized as Dipartimento di eccellenza by the Italian Ministry of University and Research.

## Conflict of Interest

The authors declare that the research was conducted in the absence of any commercial or financial relationships that could be construed as a potential conflict of interest. The reviewer SM declared a shared affiliation with the authors to the handling editor at the time of the review.

## Publisher's Note

All claims expressed in this article are solely those of the authors and do not necessarily represent those of their affiliated organizations, or those of the publisher, the editors and the reviewers. Any product that may be evaluated in this article, or claim that may be made by its manufacturer, is not guaranteed or endorsed by the publisher.

## References

[B1] Ayers E. I. Tow A. C. Holtzer R. Verghese J. (2014). Walking while talking and falls in aging. Gerontology 60, 108–113. 10.1159/000355119 24192342 PMC3944080

[B2] Ball K. Edwards J. Ross L. A. McGwin G Jr. (2010). Cognitive training decreases motor vehicle collision involvement of older drivers. J. Am. Geriatr. Soc. 58, 2107–2113. 10.1111/j.1532-5415.2010.03138.x 21054291 PMC3057872

[B3] Ball K. Owsley C. (1993). The useful field of view test: a new technique for evaluating age-related declines in visual function. J. Am. Optom. Assoc. 64, 71–79. 8454831

[B4] Bartolomeo P. de Schotten M. T. Chica A. B. (2012). Brain networks of visuospatial attention and their disruption in visual neglect. Front. Hum. Neurosci. 6:110. 10.3389/fnhum.2012.00110 22586384 PMC3343690

[B5] Beauchet O. Annweiler C. Dubost V. Allali G. Kressig R. W. Bridenbaugh S.. (2009). Stops walking when talking: a predictor of falls in older adults? Eur. J. Neurol. 16, 786–795. 10.1111/j.1468-1331.2009.02612.x 19473368

[B6] Bonato M. (2012). Neglect and extinction depend greatly on task demands: a review. Front. Hum. Neurosci. 6, 1–13. 10.3389/fnhum.2012.00195 22822394 PMC3398353

[B7] Bonato M. (2015). Unveiling residual, spontaneous recovery from subtle hemispatial neglect three years after stroke. Front. Hum. Neurosci. 9:413. 10.3389/fnhum.2015.00413 26283942 PMC4519683

[B8] Bonato M. Romeo Z. Blini E. Pitteri M. Durgoni E. Passarini L.. (2019). Ipsilesional impairments of visual awareness after right-hemispheric stroke. Front. Psychol. 10:697. 10.3389/fpsyg.2019.00697 31024378 PMC6465520

[B9] Clay O. J. Wadley V. G. Edwards J. D. Roth D. L. Roenker D. L. Ball K. K. (2005). Cumulative meta-aanalysis of the relationship between useful field of view and driving performance in older adults: current and future implications. Optom. Vis. Sci. 82, 724–731. 10.1097/01.opx.0000175009.08626.65 16127338

[B10] Corbetta M. Shulman G. L. (2002). Control of goal-directed and stimulus-driven attention in the brain. Nat. Rev. Neurosci. 3, 201–215. 10.1038/nrn755 11994752

[B11] Craik F. M. Bialystok E. (2006). Planning and task management in older adults: cooking breakfast. Mem. Cogn. 34, 1236–1249. 10.3758/BF03193268 17225505

[B12] Cross J. M. McGwin G. Rubin G. S. Ball K. K. West S. K. Roenker D. L.. (2009). Visual and medical risk factors for motor vehicle collision involvement among older drivers. Br. J. Ophthalmol. 93, 400–404. 10.1136/bjo.2008.144584 19019937 PMC2747632

[B13] De Angelis T. (2009). Older adults in the driver's seat. Monit. Psychol. 40:52.

[B14] De Lucia N. Grossi D. Mauro A. Trojano L. (2015). Closing-in in Parkinson's disease individuals with dementia: an experimental study. J. Clin. Exp. Neuropsychol. 37, 946–955. 10.1080/13803395.2015.1071339 26332174

[B15] Driver J. Vuilleumier P. (2001). Perceptual awareness and its lossi unilateral neglect and extinction. Cognition 79, 39–88. 10.1016/S0010-0277(00)00124-4 11164023

[B16] Edwards J. D. Myers C. Ross L. A. Roenker D. L. Cissell G. M. McLaughlin A. M.. (2009). The longitudinal impact of cognitive speed of processing training on driving mobility. Gerontologist 49, 485–494. 10.1093/geront/gnp042 19491362 PMC2709540

[B17] Edwards J. D. Ross A. L. Wadley V. G. Clay O. J. Crowe M. Roenker D. L.. (2006). The useful field of view test: normative data for older adults. Arch. Clin. Neuropsychol. 21, 275–286. 10.1016/j.acn.2006.03.001 16704918

[B18] Harada C. N. Natelson Love M. C. Triebel K. (2013). Normal cognitive aging. Clin. Geriatr. Med. 29, 737–752. 10.1016/j.cger.2013.07.002 24094294 PMC4015335

[B19] Howard Z. L. Evans N. J. Innes R. J. Brown S. D. Eidels A. (2020). How is multitasking different from increased difficulty? Psychon. Bull. Rev. 27, 937–951. 10.3758/s13423-020-01741-8 32440999

[B20] Kannus P. Sievänen H. Palvanen M. Järvinen T. Parkkari J. (2005). Prevention of falls and consequent injuries in elderly people. Lancet 366, 1885–1893. 10.1016/S0140-6736(05)67604-0 16310556

[B21] Kessel M. E. Geurts A. C. H. Brouwer W. H. Fasotti L. (2013). Visual scanning training for neglect after stroke with and without a computerized lane tracking dual task. Front. Hum. Neurosci. 7:358. 10.3389/fnhum.2013.00358 23847519 PMC3707289

[B22] Leone C. Patti F. Feys P. (2015). Measuring the cost of cognitive-motor dual tasking during walking in multiple sclerosis. Mult. Scler. J. 21, 123–131. 10.1177/1352458514547408 25178543

[B23] Lundin-Olsson L. Nyberg L. Gustafson Y. (1997). Stops walking when talking as a predictor of falls in elderly people. Lancet 349:9617. 10.1016/S0140-6736(97)24009-2 9057736

[B24] Montero-Odasso M. M. Sarquis-Adamson Y. Speechley M. Borrie M. J. Hachinski V. C. Wells J.. (2017). Association of dual-task gait with incident dementia in mild cognitive impairment. JAMA Neurol. 74:857. 10.1001/jamaneurol.2017.0643 28505243 PMC5710533

[B25] Nielsen M. S. Simonsen A. H. Siersma V. Hasselbalch S. G. Hoegh P. (2018). The diagnostic and prognostic value of a dual-tasking paradigm in a memory clinic. J. Alzheimer's Dis. 61, 1189–1199. 10.3233/JAD-161310 29278887

[B26] Oliva H. N. P. Mansur Machado F. S. Rodrigues V. D. Leão L. L. Monteiro-Júnior R. S. (2020). The effect of dual-task training on cognition of people with different clinical conditions: an overview of systematic reviews. IBRO Neurosci. Rep. 9, 24–31. 10.1016/j.ibror.2020.06.005 33336101 PMC7733129

[B27] Rengachary J. D'Avossa G. Sapir A. Shulman G. L. Corbetta M. (2009). Is the posner reaction time test more accurate than clinical tests in detecting left neglect in acute and chronic stroke? Arch. Phys. Med. Rehabil. 90, 2081–2088. 10.1016/j.apmr.2009.07.014 19969172 PMC3755360

[B28] Rubin G. S. Edmond S. W. Ng Bandeen-Roche K. Keyl P. M. Freeman E. E.. (2007). A prospective, population-based study of the role of visual impairment in motor vehicle crashes among older drivers: the SEE study. Investig. Ophthalmol. Vis. Sci. 48:1483. 10.1167/iovs.06-0474 17389475

[B29] Schaefer S. (2014). The ecological approach to cognitive–motor dual-tasking: findings on the effects of expertise and age. Front. Psychol. 5:1167. 10.3389/fpsyg.2014.01167 25352820 PMC4196472

[B30] Sekuler B. A. Patrick J. B. Mamelak M. (2000). Effects of aging on the useful field of view. Exp. Aging Res. 26:103–120. 10.1080/036107300243588 10755218

[B31] Strouwen C. Molenaar E. Münks L. Keus S. Bloem B. Rochester L.. (2015). Dual tasking in parkinson's disease: should we train hazardous behavior? Expert Rev. Neurother. 15, 1031–1039. 10.1586/14737175.2015.1077116 26289490

[B32] Van Vleet T. M. DeGutis J. M. (2013). Cross-training iìin hemispatial neglect: auditory sustained attention training ameliorates visual attention deficits. Cortex 49, 679–690. 10.1016/j.cortex.2012.03.020 22578712

[B33] Verghese J. Buschke H. Viola L. Katz M. Hall C. Kuslansky G.. (2002). Validity of divided attention tasks in predicting falls in older individuals: a preliminary study. J. Am. Geriatr. Soc. 50, 1572–1576. 10.1046/j.1532-5415.2002.50415.x 12383157

[B34] Vuilleumier P. (2013). Mapping the functional neuroanatomy of spatial neglect and human parietal lobe functions: progress and challenges. Ann. N. Y. Acad. Sci. 1296, 50–74. 10.1111/nyas.12161 23751037

[B35] Wang M. Y. Chang C. Y. Su S. Y. (2011). What is cooking? cognitive training of executive function in the elderly. Front. Psychol. 2:228. 10.3389/fpsyg.2011.00228 21954388 PMC3173828

[B36] Wood J. M. Anstey K. J. Kerr G. K. Lacherez P. F. Lord S. (2008). A multidomain approach for predicting older driver safety under in-traffic road conditions. J. Am. Geriatr. Soc. 56, 986–993. 10.1111/j.1532-5415.2008.01709.x 18422946

[B37] Wood J. M. Horswill M. S. Lacherez P. F. Anstey K. J. (2013). Evaluation of screening tests for predicting older driver performance and safety assessed by an on-road test. Accid. Anal. Prev. 50, 1161–1168. 10.1016/j.aap.2012.09.009 23089560

[B38] Wood J. M. Owsley C. (2014). Gerontology viewpoint: useful field of view test. Gerontology 60, 315–318. 10.1159/000356753 24642933 PMC4410269

[B39] Yogev-Seligmann G. Giladi N. Brozgol M. Hausdorff J. M. (2012). A training program to improve gait while dual tasking in patients with Parkinson's disease: a pilot study. Arch. Phys. Med. Rehabil. 93, 176–181. 10.1016/j.apmr.2011.06.005 21849167

